# Strategic technological innovation through ChatMu: transforming information accessibility in Muhammadiyah

**DOI:** 10.3389/frai.2025.1446590

**Published:** 2025-02-04

**Authors:** Muhammad Syahriandi Adhantoro, Dedi Gunawan, Harun Joko Prayitno, Rahayu Febri Riyanti, Eko Purnomo, Adi Jufriansah

**Affiliations:** ^1^Faculty of Communication and Informatics, Universitas Muhammadiyah Surakarta, Surakarta, Indonesia; ^2^Faculty of Teacher Training and Education, Universitas Muhammadiyah Surakarta, Surakarta, Indonesia; ^3^Faculty of Teacher Training and Education, Universitas Muhammadiyah Maumere, Maumere, Indonesia

**Keywords:** artificial intelligence, ChatMu application, chatbot technology, digital literacy, Muhammadiyah, system usability scale, user engagement

## Abstract

This study examines the effectiveness of the ChatMu application in improving access to information for members of Muhammadiyah, a prominent socio-religious organization. The research employs a mixed-methods approach, combining qualitative and quantitative analyses to evaluate the application’s performance, usability, and user satisfaction. Findings reveal that ChatMu significantly enhances the accessibility and accuracy of Muhammadiyah-related information, highlighting its potential as an innovative tool for addressing community-specific information needs. However, several usability challenges were identified, including navigation inefficiencies and inconsistencies in content delivery. These limitations suggest the need for further refinement to optimize user experience and functionality. Despite these issues, ChatMu demonstrates strong capabilities in providing relevant and reliable information, fostering digital literacy, and supporting information dissemination within the Muhammadiyah community. The study concludes that ChatMu represents a promising application of chatbot technology in empowering communities through improved access to knowledge. Future development efforts should focus on comprehensive usability testing, maintaining information relevance, and incorporating advanced interactive features to enhance engagement. With continuous improvements, ChatMu has the potential to become an effective medium for advancing literacy and knowledge-sharing in the Muhammadiyah community.

## 1 Introduction

Members of Muhammadiyah currently face significant challenges in accessing information related to the organization’s activities and issues. Despite being a large organization with an extensive network, limitations in information accessibility remain a major obstacle for many members. One of the primary contributing factors is the insufficient conversion of information into digital formats (Okan, 2021). Most information about Muhammadiyah is still available in conventional forms, such as printed books, magazines, and other physical materials, which are not easily accessible to all members (Fanani et al., 2021). Furthermore, despite the rapid advancements in information technology, a considerable portion of Muhammadiyah-related information remains unavailable in digital formats.

This situation is exacerbated by the disparity in internet access across Indonesia. While urban areas generally enjoy relatively stable and comprehensive internet access, many rural regions suffer from limited or even non-existent internet coverage. This digital divide not only hampers access to digital resources for members in rural areas but also restricts their ability to access conventional materials that often require robust distribution networks (Ariaji et al., 2021; Berardi et al., 2023).

Addressing these challenges necessitates innovative and effective solutions. One promising approach is the introduction of ChatMu, an artificial intelligence (AI)-powered chatbot specifically designed to facilitate access to information about Muhammadiyah. ChatMu is tailored to meet the needs and preferences of its users, with WhatsApp selected as its primary platform due to its widespread use and accessibility.

Previous studies have highlighted the persistent issues in accessing Muhammadiyah-related information. Research by Hariyadi and Noviliza (2021) revealed that many members struggle to access information about Muhammadiyah’s teachings and values, particularly in rural areas with limited internet connectivity. Similarly, Pasaribu and Suyanto (2020) emphasized that the lack of digitization of information remains a significant barrier to improving literacy among Muhammadiyah members. Moreover, Burhani’s (2018) study identified that most Muhammadiyah-related information is still disseminated through conventional formats such as printed books and magazines, which are often less accessible to younger generations who prefer digital platforms.

The choice of WhatsApp as the foundation for ChatMu is strategic, given its pervasive popularity in Indonesia. As one of the most widely used communication platforms in the country, WhatsApp offers an intuitive and user-friendly interface, making it an ideal medium for delivering information services to Muhammadiyah members (Ortiz et al., 2018; Alamer and Al Khateeb, 2023). Additionally, WhatsApp’s compatibility with various devices, including smartphones commonly used by Indonesians, ensures broad accessibility.

The development of ChatMu represents a significant advancement in overcoming the information access challenges faced by Muhammadiyah members, particularly in relation to organizational issues. It also contributes to academic discourse by demonstrating the application of modern technologies to address specific societal needs. Research by Borsci et al. (2022) underscores the pivotal role of technological innovation in enhancing information accessibility, especially for communities with unique needs, such as Muhammadiyah members. In this context, ChatMu not only offers a readily accessible source of information but also fosters an interactive platform for knowledge sharing and experience exchange among members.

A study by Cheng and Jiang (2022) further supports the notion that AI-driven chatbots can significantly enhance efficiency in information dissemination and service delivery. Consequently, the integration of ChatMu with the WhatsApp platform not only addresses the demand for rapid and accurate information access but also exemplifies the potential of technology in religious and cultural contexts.

To provide a comprehensive solution to the issue of information accessibility among Muhammadiyah members, this research also considers the accessibility disparities between urban and rural areas. Zhou et al. (2020) argue that leveraging information and communication technology, such as mobile applications, can bridge the information access gap between urban and rural communities. Thus, ChatMu is envisioned not merely as an information tool but as an inclusive and sustainable technological innovation for the Muhammadiyah community.

Ensuring the success of ChatMu’s implementation requires attention to several critical factors. First, the development of a comprehensive and reliable content database is essential for delivering high-quality and relevant information. The information provided through ChatMu must be accurate, dependable, and aligned with the needs and expectations of its users (Khan and Albatein, 2021). Second, adequate training and support must be provided to members to enable them to utilize ChatMu effectively (Nobre et al., 2020). With proper training, members are expected to adapt quickly to the use of this technology.

In the long term, sustaining ChatMu’s development and operations will necessitate the ongoing commitment and support of various stakeholders, including Muhammadiyah officials, members, technology developers, and AI experts (Gomaa et al., 2023). With strong collaboration from all parties involved, ChatMu has the potential to become an effective solution for improving access to Muhammadiyah-related information and literacy while contributing to the organization’s growth as a progressive and dynamic Islamic movement (Putri et al., 2022).

Based on the considerations outlined above, this study seeks to answer critical questions: whether all Muhammadiyah members can easily and efficiently access information through ChatMu, how accurate and reliable the responses provided by ChatMu are, and whether the information shared by ChatMu is consistently up-to-date.

## 2 Methodology

This study adopts the ADDIE model as its methodological framework, encompassing the stages of Analysis, Design, Development, Implementation, and Evaluation. The ADDIE model is widely utilized in the development of educational programs and technological solutions, particularly those emphasizing user-centered designs (Syahmani et al., 2021). Below is a detailed explanation of how the ADDIE framework is applied in this research:

### 2.1 Analysis

The first phase involves conducting an in-depth analysis of the challenges faced by Muhammadiyah members in accessing information related to Muhammadiyah affairs. This includes assessing the current level of Muhammadiyah literacy, identifying key factors influencing information accessibility, and understanding user preferences and needs for information retrieval. To support this analysis, the primary information sources integrated into the ChatMu application are detailed in Table 1. These sources include official Muhammadiyah publications, archival news data, and academic references, ensuring that the application is built on a robust foundation. The analysis also informs the customization of ChatMu’s features to align with user requirements.

**Table 1:** Database ChatMu.

Information source	Description
The Book of “Himpunan Putusan Tarjih Muhammadiyah”	Is an official document issued by the Tarjih and Tajdid PP Muhammadiyah Assemblies. This book contains the decisions derived from the National (Munas) Tarjih Decision. The Tarjah Decision occupies the strongest position in the hierarchy of the decisions made by the tarjih and tajdid assemblies (Iqbal and Suyono 2018).
Book of “Tanya Jawab Agama”	It’s a collection of hundreds of questions asked by citizens from all walks of life. The questions were answered by the Tarjih and Tajdid Assemblies and covered various religious themes. To date, there are at least eight editions of the book “Tanya Jawab Agama” that have been published (Rachmadhani et al., 2022).
Book of “Al-Islam dan Kemuhammadiyahan”	Is a textbook prepared to provide students with insight into the basic material of Muhammadiyah (Lahmi et al., 2022).
Suara Muhammadiyah Magazine and Tabloid	Is one of the sources of Muhammadiyah da’wah which is printed and distributed to all Muhammadiyah residents to find out the latest news about Muhammadiyah, and the Suara Muhammadiyah website is a website which contains news or news or all activities about Muhammadiyah (Zara, 2022).
Tanfidz Muktamar	The official publication issued by Muhammadiyah after the holding of Muktamar, a major conference held by the organization on a periodic basis. Muktamar Muhammadiyah is the highest forum in the organizational structure, where members from all over Indonesia gather to discuss and define policies, programmes of work, and organizational strategies for the next period (Burhani, 2023).

### 2.2 Design

**Figure 1:**
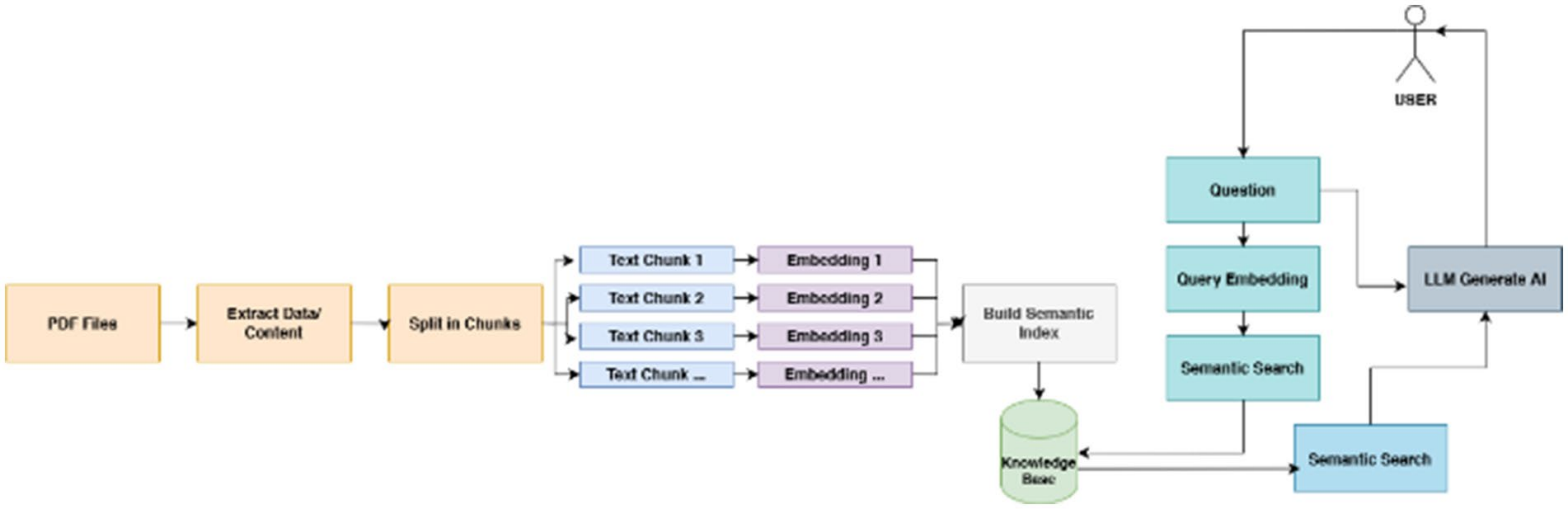
Explains the structure of the application development process and its elements.

Building on the analysis, the Design phase focuses on conceptualizing a solution tailored to user needs and characteristics (Ma and Zhang, 2021). The design stage encompasses the creation of ChatMu’s functionalities and features. Key activities include developing an intuitive user interface, structuring an efficient database system, and planning effective strategies for delivering content to users. The overall design and architecture of the ChatMu application are visually presented in Figure 1. This phase emphasizes usability and accessibility, ensuring the system meets the expectations of Muhammadiyah members.

### 2.3 Development

**Figure 2:**
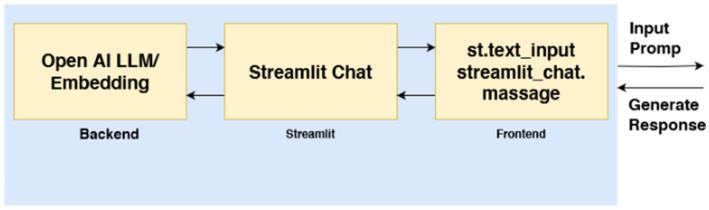
Explains the system reading flow based on the prompt provided by the user.

The Development phase involves transforming the conceptual design into a functional application. ChatMu is developed using the Python programming language, with the PyPDF2 library utilized for processing PDF-format database files (Anki and Bustamam, 2021), as outlined in Table 1. Server endpoints are then created to execute the designed functionalities, enabling seamless communication between the application and its backend systems. These endpoints are subsequently integrated into the WhatsApp platform, chosen for its widespread adoption in Indonesia, particularly among Muhammadiyah members (Mendoza et al., 2023). WhatsApp provides a familiar and accessible platform for users, ensuring broad applicability. During this phase, iterative testing and refinement are performed to ensure ChatMu operates effectively and adheres to quality standards. The detailed operational workflow of ChatMu is depicted in Figure 2.

### 2.4 Implementation

Upon completing development, the application enters the Implementation phase. Before its official rollout, ChatMu undergoes rigorous testing using the black-box testing method, which evaluates the system’s external functionalities without examining the internal code structure (Huynh et al., 2019). Black-box testing ensures the accuracy of ChatMu’s responses to user queries, its stability, responsiveness to various inputs, and seamless integration with WhatsApp (Sharma et al., 2021). Based on the results of these tests, necessary adjustments and refinements are made to optimize the application’s performance. After successful testing, ChatMu is introduced to Muhammadiyah members for widespread usage.

### 2.5 Evaluation

The final stage, Evaluation, assesses the efficacy of ChatMu in enhancing access to information about Muhammadiyah affairs. This phase involves gathering data on user satisfaction, improvements in Muhammadiyah literacy, and the overall impact of ChatMu’s implementation. To evaluate user experience, questionnaires are distributed using the System Usability Scale (SUS) method. SUS is a widely recognized tool for measuring the usability and user experience of applications (Lewis, 2018). Users are asked to rate various aspects of ChatMu, such as its ease of use, functionality, efficiency, and overall satisfaction (Thamilarasan et al., 2023). The results from the SUS evaluation provide insights into the system’s strengths and areas for improvement. These findings guide future enhancements to ChatMu, ensuring its sustainability and alignment with user needs. The evaluation also lays the groundwork for subsequent developments and serves as a reference for the design of similar solutions in the future.

By employing the ADDIE framework, this study ensures a systematic and iterative approach to developing ChatMu, ultimately addressing the information access challenges faced by Muhammadiyah members.

## 3 Result and discussion

Before implementing the ChatMu application for users, an important step is to conduct blackbox testing to ensure that all features and functions can run smoothly. Blackbox testing is conducted as part of the application development process to validate system performance from the user’s perspective without considering the internal structure or code of the application (Dahito et al., 2023). According to Darla (2021) with blackbox testing, developers can identify and fix potential bugs or issues in the application before it is presented to end users. This is crucial to ensure that the user experience with the ChatMu application is positive and meets expectations. Testing using blackbox testing is illustrated in Table 2.

**Table 2:** ChatMu blackbox test results.

Tested features	Input	Expected output	Test result
Introduction initial greetings	“Assalamualaikum”	Response from the system	Valid
Introduction initial greetings	“Hi”	Response from the system	Valid
Introduction to questions	“Apa itu Muhammadiyah?”	Relevant answers about Muhammadiyah	Valid
Introduction to questions	“What is Muhammadiyah?”	Relevant answers about Muhammadiyah	Valid
Use of Bahasa Indonesia	Questions in Bahasa Indonesia	Correct answer in Bahasa Indonesia	Valid
Use of English	Questions in English	Correct answer in English	Valid
Consistency of answers	Same question in different languages	The answer is the same and consistent	Valid

Based on the results of blackbox testing conducted on the ChatMu application in Table 2, this application has successfully passed a series of tests with satisfactory results. This aligns with the research by Kirinuki and Tanno (2024) which indicates that large language models like ChatGPT can produce test cases that generally match or slightly exceed test cases created by human participants in terms of test coverage. Key features such as initial greeting recognition, question recognition, and the use of both Indonesian and English languages have performed well and provided outputs as expected. The use of different languages also did not affect the consistency of answers provided by the application, indicating the application’s ability to respond well to user requests in various language contexts. This demonstrates that the ChatMu application has been well-designed and developed to meet the needs and preferences of users in accessing information about Muhammadiyah.

The results of blackbox testing provide confidence that the ChatMu application is ready to be implemented for users with reliable and consistent performance. Other research by Pavlik (2023) also indicates that large language models like ChatGPT have impressive capabilities in various applications, including essay writing and programming, which supports the results of blackbox testing.

**Figure 3:**
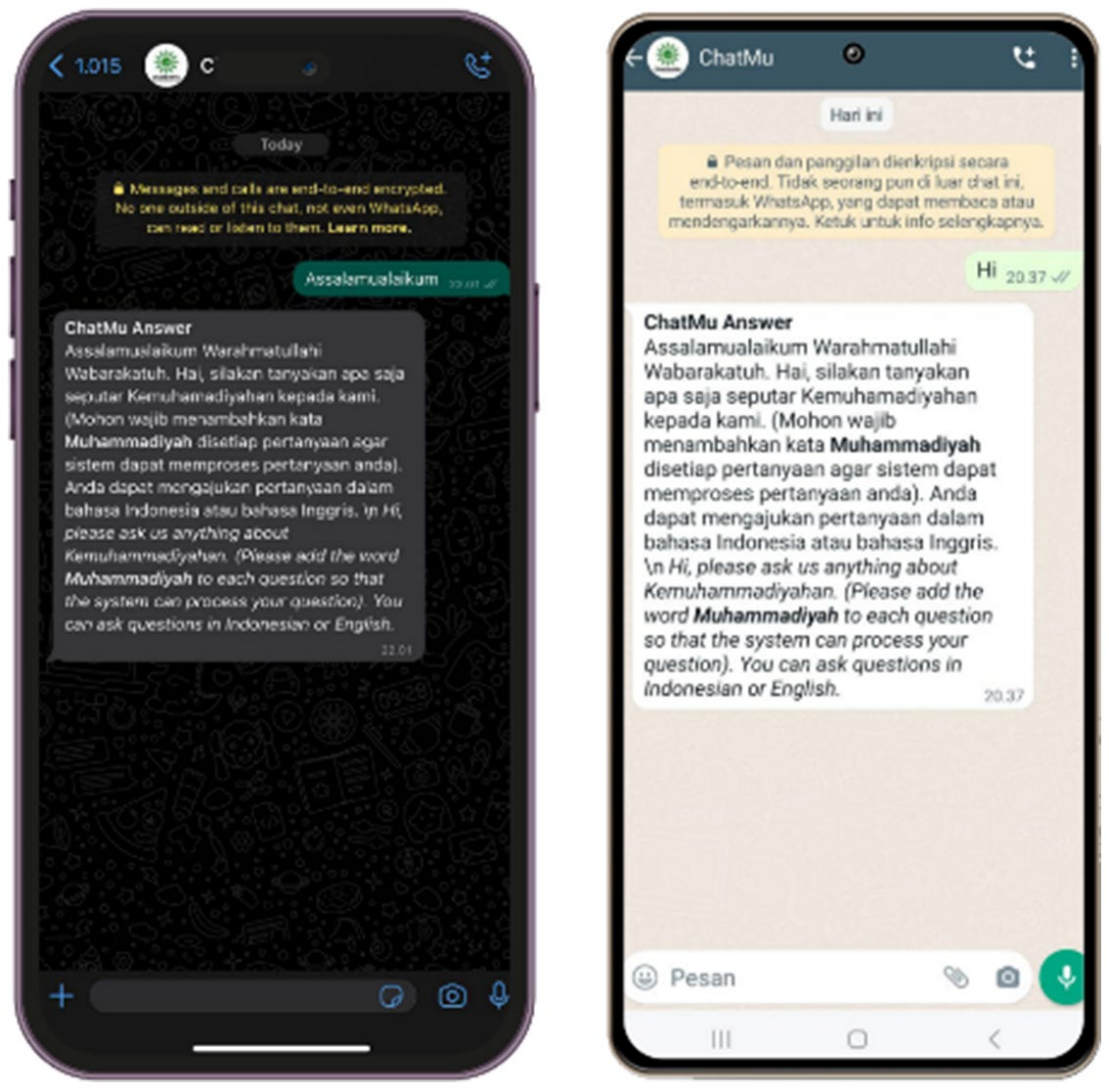
Explains the processing results of the system’s responses based on the user’s prompt.

The use of the ChatMu application seems quite straightforward; users only need to enter the designated number. From the provided description, the process of using the application appears to be quite simple and intuitive. The first step users take is to greet, such as “Assalamualaikum” or “Hi,” before asking questions. Subsequently, the system provides a welcome response along with instructions for users to ask questions related to Muhammadiyah by inserting the word “Muhammadiyah” in their questions. This step helps the system to provide more accurate and relevant answers to the intended topic. This process is illustrated in Figure 3.

Based on Figure 3, it can be concluded that the ChatMu system is capable of responding well and appropriately to initial greetings from users, as expected. The following are the initial response results from the system after users greet.

Assalamualaikum Warahmatullahi Wabarakatuh. *Hai, silakan tanyakan apa saja seputar Kemuhamadiyahan kepada kami. (Mohon wajib menambahkan kata Muhammadiyah disetiap pertanyaan agar sistem dapat memproses pertanyaan anda). Anda dapat mengajukan pertanyaan dalam bahasa Indonesia atau bahasa Inggris.* \n Hi, please ask us anything about Kemuhammadiyahan. (Please add the word Muhammadiyah to each question so that the system can process your question). You can ask questions in Indonesian or English.

**Figure 4:**
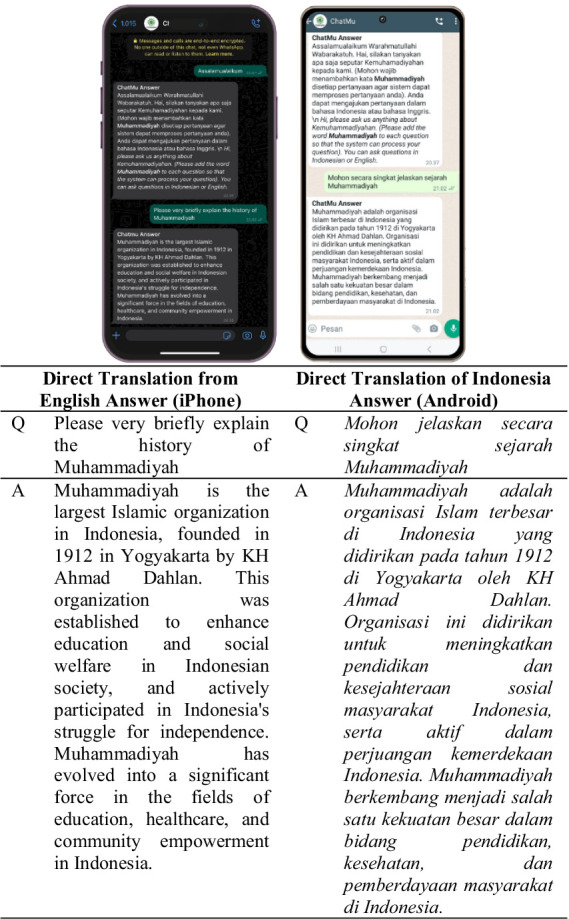
ChatMu system response in answering questions from users.

In addition to ease of use, the ChatMu application also supports the use of two languages, namely Indonesian and English. Users can ask questions in their preferred language, thereby expanding the usage of this application to various segments of society, including those who may not be fluent in the Indonesian language. As seen in Figure 4, users use two different devices, namely devices with Android and iPhone operating systems, with the aim of comparing the accuracy and consistency of the answers provided by the application.

Testing on the first question, users ask questions about an explanation of the history of Muhammadiyah, as seen in Figure 4. Looking at the results presented in Figure 4, on both devices with Android and iPhone operating systems, the questions asked are the same but in different languages. However, the answer results from both devices are the same, indicating the consistency and accuracy of the answers provided by the application, even though the questions are asked in different languages. This demonstrates the ChatMu application’s ability to understand and respond well to user requests, regardless of the language used by the user.

Testing on the second question displays a more substantive nature as it involves a more complex process for the system. In this case, the ChatMu system is challenged to do more than just respond to user requests (Khan et al., 2023). Instead, the system must be able to read, collect, and analyze the information contained in the question to provide accurate and relevant answers (Cooper, 2023). This process involves understanding the context and substance of the questions asked by users, thus requiring a higher level of processing from the system.

**Figure 5:**
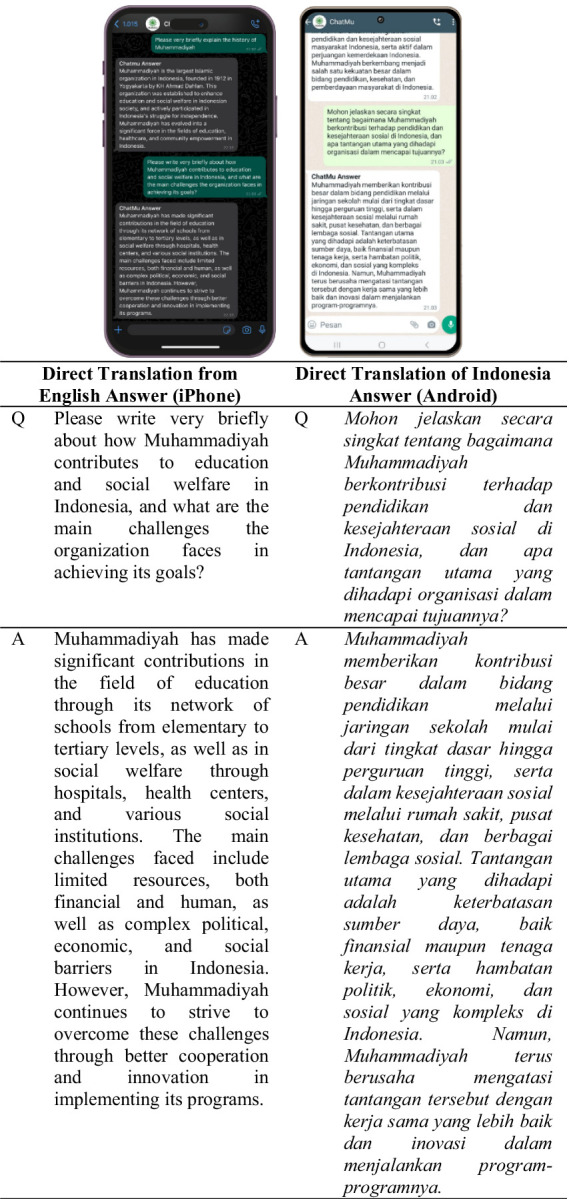
ChatMu system response in answering questions from users.

Use of the system as illustrated in Figure 5, helps to illustrate the interaction between users and the system, as well as highlighting the complexity of the questions and answers involved (Rahman and Watanobe, 2023). By visualizing this interaction, the importance of the ChatMu system’s ability to accurately understand and respond to user requests can be seen, especially when the questions require substantial analysis to provide satisfactory answers (Halaweh, 2023).

Testing on the second question provides a deeper understanding of the system’s performance and capabilities in facing more complex challenges in providing meaningful answers to users. Research by Hoque et al. (2023) shows that large language models like ChatGPT can support complex question-answering scenarios and increase audience engagement. However, other research by Bahak et al. (2023) shows that although ChatGPT demonstrates competence as a generative model, it is less effective in answering questions compared to task-specific models.

From the results presented in Figure 5, it can be concluded that the ChatMu system still demonstrates consistency in providing answers to questions asked by users, regardless of the device or language used. This consistency is reflected in the results showing that there is no significant difference between the answers provided by the system for questions asked by users via iPhone devices using English and Android devices using Indonesian (Kavaz et al., 2023). Previous research conducted by Lew et al. (2018) has also shown that in online chat-based communication, agents typically use pre-composed responses and talk to multiple customers simultaneously to increase efficiency. However, this technique can affect conversational interactivity-contingency dimensions and response latency that can undermine interpersonal assessment, satisfaction, and organizational relationships with customers. This means that the system has been able to provide consistent and relevant answers to the content of the questions asked, without discrimination based on the language or device used by the user. This is in line with future research directions in the field of chatbot, which include user experience and design, frameworks and platforms, chatbots for collaboration, chatbot democratization, as well as ethics and privacy (Følstad et al., 2021).

To reconfirm the performance of the ChatMu system, users attempt to provide more substantive and technical questions. This challenge is intended to test the system’s ability to handle more complex questions and ensure that the system can continue to function well in more challenging situations. By asking deeper questions, users can push the system’s ability to understand the context and substance of the questions asked, as well as its ability to provide relevant and meaningful answers.

**Figure 6:**
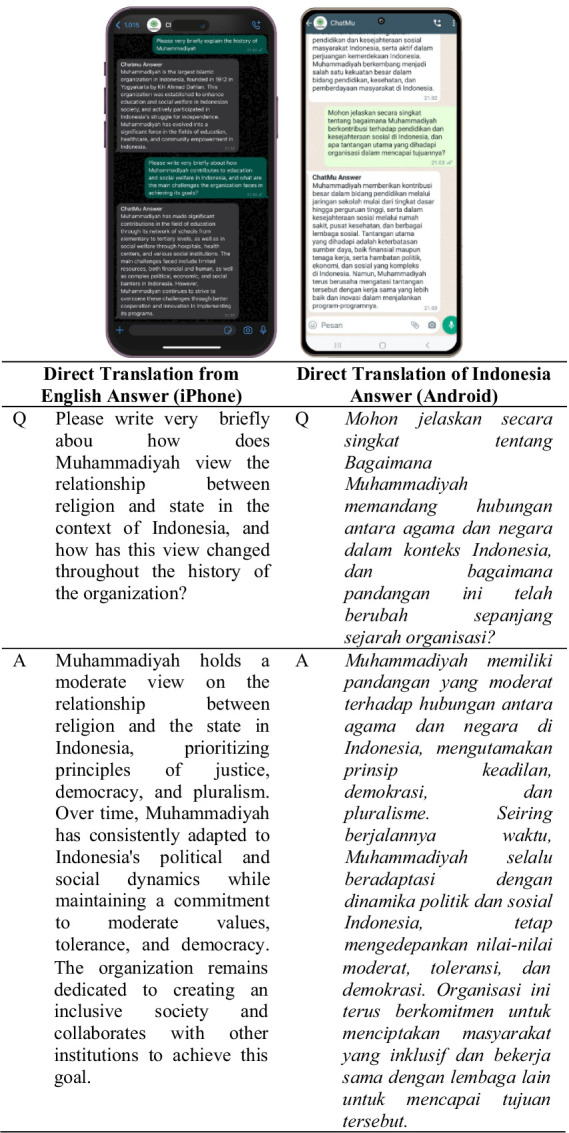
ChatMu system response in answering questions from users.

Research conducted by Carmichael et al. (2022) has shown that in online chat-based communication, agents typically use pre-composed responses and talk to multiple customers simultaneously to increase efficiency. However, this technique can affect the dimensions of interaction-contingency and response latency that can undermine interpersonal assessment, satisfaction, and organizational relationships with customers. This process also aims to test the boundaries of artificial intelligence implemented in the ChatMu system. The use of tables and Figures, as seen in Figure 6, helps to illustrate the interaction between users and the system in facing more complex questions. By visualizing this interaction, we can see how the ChatMu system responds to user requests in more challenging situations and understand the extent of the system’s ability to address these challenges.

Testing on more substantive and technical questions provides a deeper understanding of the performance and capabilities of the ChatMu system, as well as highlighting the potential and limitations of the artificial intelligence applied in this application (Helmiyah et al., 2021). This aligns with future research directions in the field of chatbots, which include user experience and design, frameworks and platforms, chatbots for collaboration, chatbot democratization, as well as ethics and privacy (Septiyanti et al., 2024).

Based on the response results provided by the ChatMu system in Figure 6, it can be concluded that the presented answers remain substantive and relevant to the questions asked by users. Analysis of the interaction between users and the system indicates that answer consistency is maintained regardless of the language or device used by the user. Both the use of iPhone devices with English and Android devices with Indonesian produce uniform responses that align with the substance of the questions asked.

Previous research by de Arriba-Pérez et al. (2023) has also shown that in online chat-based communication, agents typically use pre-composed responses and talk to multiple customers simultaneously to increase efficiency. However, this technique can affect the dimensions of interaction-contingency and response latency that can undermine interpersonal assessment, satisfaction, and organizational relationships with customers (Chen et al., 2021).

This indicates that the ChatMu system is capable of understanding and responding well to various types of questions, languages, and contexts provided. Thus, the conclusion that can be drawn is that the system remains consistent in answering every user question, maintaining high standards of user experience. These results provide strong evidence of the reliability and capability of the ChatMu system in providing consistent and high-quality information services to users.

Consistency and accuracy in providing answers are crucial in building user trust in the application (Jungmann et al., 2019). With ChatMu’s ability to provide consistent and accurate answers, wherever users use the application and in whatever language questions are asked, user satisfaction and trust in the application’s quality will increase (Martin et al., 2020). This also indicates that the use of ChatMu can be a reliable and dependable solution in meeting users’ information needs related to Muhammadiyah.

Regarding content updates, the system is updated regularly to ensure the information remains relevant. However, the frequency of updates depends on the availability of new content and data from Muhammadiyah. The system is designed to incorporate new information as it becomes available, but the timeline for these updates needs to be streamlined for consistency.

After going through the previous testing stages, the next step in evaluating the performance of the ChatMu system is to use the System Usability Scale (SUS) method (Kortum and Bangor, 2013). This method aims to evaluate the usability of the system from the user’s perspective (Vlachogianni and Tselios, 2022). By distributing the SUS questionnaire, users are asked to assess various aspects of system usability, such as ease of use, clarity of instructions, and overall user satisfaction (Cheah et al., 2023). The questions from SUS are illustrated in Table 3, providing a framework for data collection and further analysis regarding ChatMu system usability.

**Table 3:** List of SUS testing method questions.

No	Question
1	I think I will use this system again
2	I find this system complicated to use
3	I find this system easy to use
4	I need help from other people or technicians in using this system
5	I feel that the system features work as they should
6	I feel there are many things that are inconsistent (not harmonious in this system)
7	I feel like others will Fig out how to use this system quickly
8	I find this system confusing
9	I feel there are no obstacles in using this system
10	I need to get used to it first before using this system

The number of respondents in the testing is 35 respondents, consisting of two types: Muhammadiyah members and the general public who are not from Muhammadiyah. The diversity of these respondents allows for a broader and more representative view of system usability from various user perspectives. By involving both groups, usability evaluation not only considers the needs and preferences of Muhammadiyah members but also takes into account the user experience from outside the Muhammadiyah environment (Liang et al., 2018). The number of respondents in the testing is 35 respondents, consisting of two types: Muhammadiyah members and the general public who are not from Muhammadiyah. The diversity of these respondents allows for a broader and more representative view of system usability from various user perspectives. By involving both groups, usability evaluation not only considers the needs and preferences of Muhammadiyah members but also takes into account the user experience from outside the Muhammadiyah environment. This is important to ensure that the ChatMu system can be accessed and used effectively by various segments of society, not limited to Muhammadiyah members alone. In a study conducted by Yanfi et al. (2020), sability testing was conducted to measure the usability level of web-based applications. The study involved several respondents and measured variables such as Learnability, Efficiency, Memorability, Errors, and Satisfaction.

In interpreting the results of SUS, there are several criteria used, including SUS Score, Grade, and Adjective rating. First, the SUS Score is the result of calculating the average score of all questions in the SUS questionnaire. This score ranges from 0 to 100, where a higher score indicates better system usability according to users (Deshmukh and Chalmeta, 2024). For example, if the system obtains a SUS Score above 80.3, this indicates that most respondents consider the system to have good usability. However, to understand the significance of this score, it is necessary to further examine the grade and Adjective rating.

Second, the grade is a category that determines how well the system performs based on the SUS Score. Usually, the grade is divided into five categories: A, B, C, D, and F. Grade A is usually given for systems with a SUS Score above 80.3, indicating that the system is rated very good by users. Grade B is given for SUS Scores between 68 and 80.3, while grade C is given for SUS Scores of 68. Grade D is given for SUS Scores between 51 and 68, while Grade F is given for SUS Scores below 51.

Third, the Adjective rating is a qualitative description of system usability based on the SUS Score and grade obtained. This Adjective rating provides further insight into how users rate the usability of the system. For example, if the system obtains a SUS Score above 80.3 with grade A, the Adjective rating given may be “Excellent,” indicating that most users are very satisfied with the usability of the system. Based on all the SUS results, they are illustrated in Table 4.

**Table 4:** Scale SUS score.

SUS Score	Grade	Adjective rating
> 80.3	A	Excellent
68–80.3	B	Good
68	C	Okay
51–68	D	Poor
< 51	F	Awful

To calculate the SUS Score, the steps are as follows:

1. Decrease 1 of the user score for statements with strange numbers (1, 3, 5, 7, 9).
2. Decrease user score from 5 for statings with integer numbers (2, 4, 6, 8, 10).
3. Summate all scores that have been adjusted.
4. Summate all scores that have been adjusted. 4. Multiply the total by 2.5 to get the final SUS Score.

Thus, the formula is:

$\mathrm{SUSScore}=\left(\overset{10}{\underset{i=1}{\sum }}\mathrm{adjustedScore}s_{i}\right)\times 2.5$

**Figure 7:**
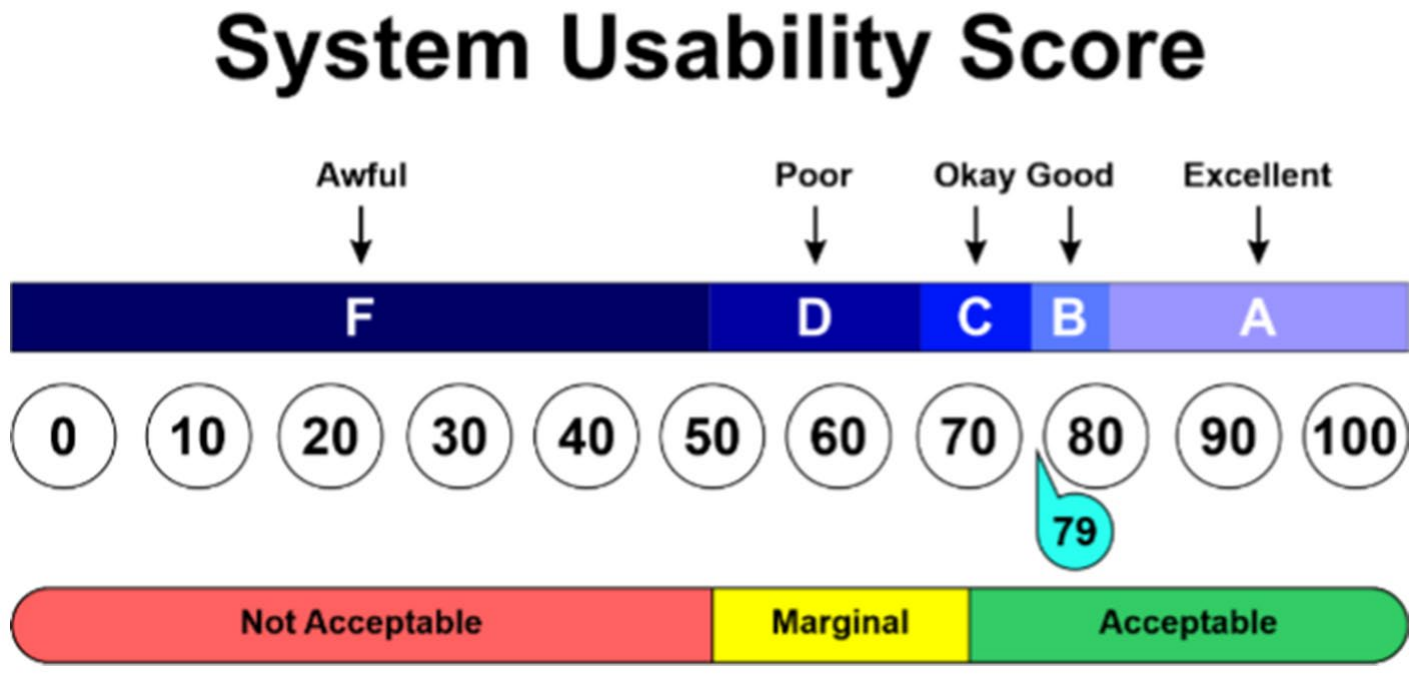
Result score of SUS.

From the System Usability Scale (SUS) testing method, it was obtained that the ChatMu application scored 79. This score falls within the range of 68–80.3, corresponding to grade B and Adjective Rating “Good.” The results of the SUS testing of the ChatMu application are illustrated in Figure 7.

The score of 79 indicates that most respondents rated the ChatMu application as a system with good usability. This score is above the threshold of 68, which is often considered as the cutoff point for acceptable usability. It indicates that most users feel that this application is easy to use, intuitive, and meets their needs in accessing information about Muhammadiyah. However, although the score is above average, there is room for improvement for the ChatMu application to achieve a higher score.

With grade B and Adjective Rating “Good,” the ChatMu application is considered to be a fairly good system according to usability standards. Grade B indicates that the application has decent usability quality but still has room for improvement (Vlachogianni and Tselios, 2022). However, the Adjective Rating “Good” depicts that users are generally satisfied with the experience of using this application. It signifies that although there are areas for improvement, the ChatMu application has provided a positive and satisfactory user experience (Derisma, 2020).

Further analysis of these results needs to look at specific aspects that influence the scores and categories given. Factors such as clarity of instructions, intuitiveness of design, response speed, and overall user satisfaction need to be further evaluated to identify areas where the application can be improved (Aljamaan et al., 2024). Additionally, user feedback also needs to be considered so that developers can identify specific issues and make appropriate improvements.

Analysis of the SUS test results for odd and even questions indicates differences in user perceptions of the usability of the ChatMu system. From odd questions, such as Q1, Q3, Q5, Q7, and Q9, relatively high scores were obtained, averaging around 132. This indicates that users tend to agree that the ChatMu system is easy to use and intuitive. The high scores on odd questions indicate that the system excels in presenting information clearly, being responsive to user requests, and providing a satisfying user experience in the initial interaction with the application (Islam et al., 2021). However, it should be noted that the score on question Q5 is slightly lower than the others, indicating that there may be some areas that need further attention in terms of clarity or consistency of information.

From even questions, such as Q2, Q4, Q6, Q8, and Q10, the scores tend to be lower, averaging around 88. This indicates that users have more negative perceptions of certain aspects of the system’s usability (Hidayat et al., 2022). For example, the low scores on questions Q8 and Q10 indicate that users may have difficulty in navigation or finding specific features in the application. Additionally, the low score on question Q4 suggests that there are issues with the clarity of instructions or guidance provided by the system.

**Figure 8:**
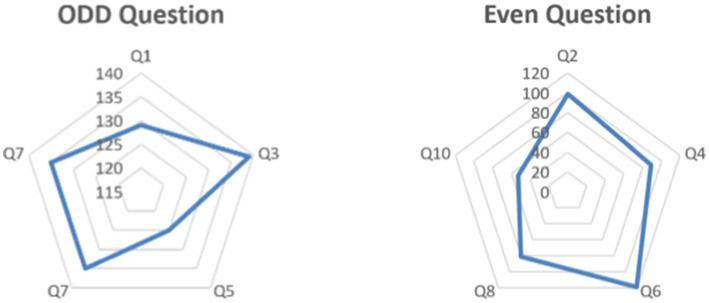
Result test of SUS.

Overall, the analysis of SUS scores indicates that the ChatMu system excels in presenting information clearly and being responsive to user requests. However, there are some weaknesses that need to be addressed, especially regarding less intuitive navigation, clarity of instructions, and consistency of information. To improve the usability of the system, further evaluation of areas with low scores and appropriate improvements based on user feedback are necessary (Cheah et al., 2023). Thus, the ChatMu system can be more effective in meeting user needs and expectations, providing a better overall user experience. The results of the SUS testing through odd and even questions are illustrated in Figure 8.

Referring to the research questions, it indicates that some aspects have been addressed through SUS testing and other evaluations of the ChatMu application. Firstly, regarding the ease and speed of accessing information about Muhammadiyah through ChatMu, the results of the SUS testing show that most users tend to agree that the system is easy to use and responsive to requests (Vlachogianni and Tselios, 2022). However, there are still some weaknesses, especially concerning less intuitive navigation and clarity of instructions. This indicates that while information accessibility may have been fulfilled for most users, there is still room for improvement to make the application more efficient and user-friendly for all Muhammadiyah members.

Secondly, regarding the accuracy of answers provided by ChatMu, SUS testing provides an overview that users are generally satisfied with the answers provided by the system. However, low scores on some even questions suggest that there is still room to improve the accuracy of answers, especially concerning consistency of information and clarity of instructions. Therefore, although most answers provided are proven accurate, further evaluation is needed to ensure that all information presented by ChatMu meets high accuracy standards (Rahmatizadeh et al., 2024).

Thirdly, regarding the availability of up-to-date information, the results of the testing do not directly indicate whether the information provided by ChatMu is up-to-date. However, as part of further evaluation, further investigation into the sources of information used by ChatMu is necessary to ensure that the information presented is always updated and relevant to the latest developments in Muhammadiyah (Afif, 2023).

Despite these improvements, the system has limitations that must be addressed in future development. One notable limitation is the restricted set of features currently offered, which could be expanded to include additional forms of media, such as images, videos, or voice responses. These types of media would enrich the user experience and could be included in future iterations of the system. Additionally, there is a need for larger-scale usability testing to identify other potential areas of improvement. Future work should also explore adding more interactive features, including personalized content recommendations and multilingual support, to further enhance accessibility and engagement.

## 4 Conclusion

This study has evaluated the effectiveness of the ChatMu application in enhancing information access for Muhammadiyah members. The findings indicate that ChatMu significantly improves the availability and accuracy of information, despite some limitations related to system navigation and consistency. Although the application has proven to be a valuable tool for meeting community-specific information needs, there are areas for further improvement. Future recommendations for the system include enhancing user experience through improved navigation and clear guidance to reduce user confusion. Additionally, incorporating more interactive features, such as multimedia content (e.g., images, videos, and voice), would further enrich the user engagement and expand the system’s capabilities. A more consistent update mechanism should also be implemented to ensure that the information remains current and reliable. Furthermore, larger-scale usability testing should be conducted to gather more comprehensive feedback and refine the system for broader user adoption. The practical implications of this research highlight the potential of chatbot technology in addressing the information needs of specific communities. By using a chatbot, organizations like Muhammadiyah can efficiently disseminate up-to-date information to their members, improving engagement and fostering a more informed community. The study suggests that similar systems could be applied to other community-based organizations, further supporting their information-sharing needs. Future work should focus on enhancing system functionality, scalability, and accessibility, ensuring the platform remains adaptable to evolving user expectations and technological advancements.

## Data Availability

The data presented in the study are included in the article/supplementary material, further inquiries can be directed to the corresponding author/s.

## References

[ref1] Afif M. H. (2023). Assessing students’ perceptions of Mobile applications usability using system usability scale. J. Comput. Sci. 19, 11–19. doi: 10.3844/jcssp.2023.11.19

[ref2] Alamer A. Al Khateeb A. (2023). Effects of using the WhatsApp application on language learners motivation: a controlled investigation using structural equation modelling. Comput. Assist. Lang. Learn. 36, 149–175. doi: 10.1080/09588221.2021.1903042

[ref3] Aljamaan F. Malki K. H. Alhasan K. Jamal A. Altamimi I. Khayat A.. (2024). ChatGPT-3.5 system usability scale early assessment among healthcare workers: horizons of adoption in medical practice. Heliyon 10:e28962. doi: 10.1016/j.heliyon.2024.e28962, PMID: 38623218 PMC11016609

[ref4] Anki P. Bustamam A. (2021). Measuring the accuracy of LSTM and BiLSTM models in the application of artificial intelligence by applying chatbot programme. Indonesian J. Electr. Eng. Comput. Sci. 23:197. doi: 10.11591/ijeecs.v23.i1.pp197-205

[ref5] Ariaji R. Nasution A. H. Dharma Harahap A. F. Abubakar F. Tuah S. Hilda L. (2021). Learning materials based on digital art student creativity in Universitas Muhammadiyah Tapanuli Selatan. J. Phys. Conf. Ser. 1764:012087. doi: 10.1088/1742-6596/1764/1/012087

[ref6] Bahak H. Taheri F. Zojaji Z. Kazemi A., Evaluating ChatGPT as a question answering system: A comprehensive analysis and comparison with existing models. (2023). Available at: http://arxiv.org/abs/2312.07592

[ref7] Berardi D. Callegati F. Giovine A. Melis A. Prandini M. Rinieri L. (2023). When operation technology meets information technology: challenges and opportunities. Future Internet 15:95. doi: 10.3390/fi15030095

[ref8] Borsci S. Malizia A. Schmettow M. van der Velde F. Tariverdiyeva G. Balaji D.. (2022). The Chatbot usability scale: the design and pilot of a usability scale for interaction with AI-based conversational agents. Pers. Ubiquit. Comput. 26, 95–119. doi: 10.1007/s00779-021-01582-9

[ref9] Burhani A. N. (2018). Pluralism, liberalism, and Islamism: religious outlook of Muhammadiyah. Studia Islamika, 433–470. doi: 10.15408/sdi.v25i3.7765

[ref10] Burhani A. N. (2023). Civilized congress: election and organization of the 48th Muktamar of Muhammadiyah in solo, Indonesia. Studia Islamika 30, 205–210. doi: 10.36712/sdi.v30i1.33378

[ref11] Carmichael L. Poirier S.-M. Coursaris C. K. Léger P. M. Sénécal S. (2022). Users’ information disclosure behaviors during interactions with Chatbots: the effect of information disclosure nudges. Appl. Sci. 12:12660. doi: 10.3390/app122412660

[ref12] Cheah W. H. Mat Jusoh N. Aung M. M. T. Ab Ghani A. Rebuan H. M. A. (2023). Mobile Technology in Medicine: development and validation of an adapted system usability scale (SUS) questionnaire and modified technology acceptance model (TAM) to evaluate user experience and acceptability of a Mobile application in MRI safety screening. Indian J. Radiol. Imaging 33, 036–045. doi: 10.1055/s-0042-1758198, PMID: 36855734 PMC9968523

[ref13] Chen J.-S. Le T.-T.-Y. Florence D. (2021). Usability and responsiveness of artificial intelligence chatbot on online customer experience in e-retailing. Int. J. Retail Distrib. Manag. 49, 1512–1531. doi: 10.1108/IJRDM-08-2020-0312

[ref14] Cheng Y. Jiang H. (2022). Customer–brand relationship in the era of artificial intelligence: understanding the role of chatbot marketing efforts. J. Prod. Brand Manag 31, 252–264. doi: 10.1108/JPBM-05-2020-2907

[ref15] Cooper G. (2023). Examining science education in ChatGPT: an exploratory study of generative artificial intelligence. J. Sci. Educ. Technol. 32, 444–452. doi: 10.1007/s10956-023-10039-y

[ref16] Dahito M.-A. Genest L. Maddaloni A. Neto J. (2023). A solution method for mixed-variable constrained blackbox optimization problems. Optim. Eng. 25, 2093–2148. doi: 10.1007/s11081-023-09874-0, PMID: 39808227

[ref17] Darla R. B., “A Blackbox failure rate prediction method for power electronic converters,” in 2021 IEEE Madras section conference (MASCON), IEEE, (2021), pp. 1–6.

[ref18] de Arriba-Pérez F. García-Méndez S. González-Castaño F. J. Costa-Montenegro E. (2023). Automatic detection of cognitive impairment in elderly people using an entertainment chatbot with natural language processing capabilities. J. Ambient. Intell. Humaniz. Comput. 14, 16283–16298. doi: 10.1007/s12652-022-03849-2, PMID: 35529905 PMC9053565

[ref19] Derisma D. (2020). The usability analysis online learning site for supporting computer programming course using system usability scale (SUS) in a university. Int. J. Interact. Mobile Technol. 14:182. doi: 10.3991/ijim.v14i09.13123

[ref20] Deshmukh A. M. Chalmeta R. (2024). Validation of system usability scale as a usability metric to evaluate voice user interfaces. PeerJ Comput. Sci. 10:e1918. doi: 10.7717/peerj-cs.1918, PMID: 38435614 PMC10909179

[ref21] Fanani A. Hamzani A. I. Khasanah N. Sofanudin A. (2021). Muhammadiyah’s Manhaj Tarjih: an evolution of a modernist approach to Islamic jurisprudence in Indonesia. HTS 77:6942. doi: 10.4102/hts.v77i4.6942

[ref22] Følstad A. Araujo T. Law E. L. C. Brandtzaeg P. B. Papadopoulos S. Reis L.. (2021). Future directions for chatbot research: an interdisciplinary research agenda. Computing 103, 2915–2942. doi: 10.1007/s00607-021-01016-7

[ref23] Gomaa S. Posey J. Bashir B. Basu Mallick A. Vanderklok E. Schnoll M.. (2023). Feasibility of a text messaging–integrated and Chatbot-interfaced self-management program for symptom control in patients with gastrointestinal Cancer undergoing chemotherapy: pilot mixed methods study. JMIR Form Res. 7:e46128. doi: 10.2196/46128, PMID: 37948108 PMC10674151

[ref24] Halaweh M. (2023). ChatGPT in education: strategies for responsible implementation. Contemp. Educ. Technol. 15:ep421. doi: 10.30935/cedtech/13036, PMID: 39684747

[ref25] Hariyadi K. H. Noviliza A. (2021). The influence of information technology and communication advancement especially smartphone on Muhammadiyah University of West Sumatera’s students year 2019. J. Phys. Conf. Ser. 1779:012083. doi: 10.1088/1742-6596/1779/1/012083

[ref49] Helmiyah S. Riadi I. Umar R. Hanif A. (2021). Speech Classification to Recognize Emotion Using Artificial Neural Network. Khazanah Informatika: Jurnal Ilmu Komputer dan Informatika, 7. doi: 10.23917/khif.v7i1.11913

[ref26] Hidayat A. Nugroho A. Nurfaizin S., “Usability evaluation on educational Chatbot using the system usability scale (SUS),” In 2022 seventh international conference on informatics and computing (ICIC), (2022), pp. 01–05.

[ref27] Hoque M. N. Mahfuz A. Kindi M. Hassan N., “Towards designing a question-answering Chatbot for online news: Understanding Questions and Perspectives,” (2023). Available at: http://arxiv.org/abs/2312.10650

[ref28] Huynh Q. T. Tran D. D. Nguyen D.-M. Ha N. H. Bui T. M. A. Nguyen P. L., “Generating test data for Blackbox testing from UML-based web engineering content and presentation models,” in Industrial networks and intelligent systems: 5th EAI international conference, INISCOM, (2019), pp. 207–219.

[ref29] Iqbal M. Suyono A., “Android based Tarjih Muhammadiyah information systems,” in IOP conference series: Materials Science and Engineering, (2018).

[ref30] Islam M. N. Khan S. R. Islam N. N. Rezwan-A-Rownok S. R. Z. Zaman S. R. (2021). A Mobile application for mental health care during COVID-19 Pandemic: development and usability evaluation with system usability scale. Adv. Intellig. Syst. Comput., 33–42. doi: 10.1007/978-3-030-68133-3_4

[ref31] Jungmann S. M. Klan T. Kuhn S. Jungmann F. (2019). Accuracy of a Chatbot (Ada) in the diagnosis of mental disorders: comparative case study with lay and expert users. JMIR Form. Res. 3:e13863. doi: 10.2196/13863, PMID: 31663858 PMC6914276

[ref32] Kavaz E. Puig A. Rodríguez I. (2023). Chatbot-based natural language interfaces for data visualization: a scoping review. Appl. Sci. 13:7025. doi: 10.3390/app13127025

[ref33] Khan N. A. Albatein J., “COVIBOT- an intelligent WhatsApp based advising bot for Covid-19,” In 2021 international conference on computational intelligence and knowledge economy (ICCIKE), (2021), pp. 418–422.

[ref34] Khan R. A. Jawaid M. Khan A. R. Sajjad M. (2023). ChatGPT - reshaping medical education and clinical management. Pak. J. Med. Sci. 39, 605–607. doi: 10.12669/pjms.39.2.7653, PMID: 36950398 PMC10025693

[ref35] Kirinuki H. Tanno H., “ChatGPT and human synergy in black-box testing: a comparative analysis,” (2024). Available at: http://arxiv.org/abs/2401.13924

[ref36] Kortum P. T. Bangor A. (2013). Usability ratings for everyday products measured with the system usability scale. Int. J. Hum. Comput. Interact. 29, 67–76. doi: 10.1080/10447318.2012.681221

[ref37] Lahmi A. Ritonga M. Raviusman R. Imran Y. (2022). Self control counseling for students during Covid-19 through Al-Islam and Kemuhammadiyahan curriculum. J. Curricul. Teach. 11:35. doi: 10.5430/jct.v11n2p35

[ref38] Lew Z. Walther J. B. Pang A. Shin W. (2018). Interactivity in online chat: conversational contingency and response latency in computer-mediated communication. J. Comput. Mediat. Commun. 23, 201–221. doi: 10.1093/jcmc/zmy009

[ref39] Lewis J. R. (2018). The system usability scale: past, present, and future. Int. J. Hum. Comput. Interact. 34, 577–590. doi: 10.1080/10447318.2018.1455307

[ref40] Liang J. Xian D. Liu X. Fu J. Zhang X. Tang B.. (2018). Usability study of mainstream wearable fitness devices: feature analysis and system usability scale evaluation. JMIR Mhealth Uhealth 6:e11066. doi: 10.2196/11066, PMID: 30409767 PMC6250954

[ref41] Ma W. Zhang L., “The development and practice of course building information modeling based on ADDIE model,” In 2021 3rd international conference on internet technology and educational Informization (ITEI), IEEE, (2021), pp. 248–251.

[ref42] Martin A. Nateqi J. Gruarin S. Munsch N. Abdarahmane I. Zobel M.. (2020). An artificial intelligence-based first-line defence against COVID-19: digitally screening citizens for risks via a chatbot. Sci. Rep. 10:19012. doi: 10.1038/s41598-020-75912-x, PMID: 33149198 PMC7643065

[ref43] Mendoza M. D. Hutajulu O. Y. Fibriasari H. (2023). The utilization of artificial intelligence based Chatbot in interactive learning media. J. Eng. Educ. Transf. 37, 174–188. doi: 10.16920/jeet/2023/v37i2/23159

[ref44] Nobre G. P. Ferreira C. H. G. Almeida J. M., “Beyond groups: uncovering dynamic communities on the WhatsApp network of information dissemination,” in Social informatics: 12th international conference, SocInfo (2020), pp. 252–266.

[ref45] Okan B. (2021). Are we transformed to confused decision-makers? The impact of digital and conventional media on the health-relevant choice and information overload. Int. J. Media Inform. Lit 6:259. doi: 10.13187/ijmil.2021.2.259

[ref46] Ortiz C. Ortiz-Peregrina S. Castro J. J. Casares-López M. Salas C. (2018). Driver distraction by smartphone use (WhatsApp) in different age groups. Accid. Anal. Prev. 117, 239–249. doi: 10.1016/j.aap.2018.04.018, PMID: 29723735

[ref47] Pasaribu K. A. Suyanto W. (2020). The effect of STEM-based (science, technology, engineering, and mathematics) learning model toward the students’ mathematical problem-solving ability in SD Muhammadiyah Condongcatur, Yogyakarta. J. Phys. Conf. Ser. 1511:012103. doi: 10.1088/1742-6596/1511/1/012103

[ref48] Pavlik J. V. (2023). Collaborating with ChatGPT: considering the implications of generative artificial intelligence for journalism and media education. Journal. Mass Commun. Educ. 78, 84–93. doi: 10.1177/10776958221149577

[ref50] Putri A. H. Samsudin A. Purwanto M. G. Suhandi A. (2022). Examination of conceptual change research over A decade: a bibliometric analysis using science mapping tool. Indonesian Journal on Learning and Advanced Education (IJOLAE), 4, 171–190. doi: 10.23917/ijolae.v4i3.18249

[ref51] Rachmadhani F. Mochammad Sahid M. Mokhtar A. W. (2022). Implementation of the ISLAMIC law Transformation’s rule (TAGHAYYUR AHKĀM) during COVID-19 PANDEMIC in the perspective of MAJELIS TARJIH MUHAMMADIYAH in Indonesia. Malaysian J. Syariah Law 10, 108–117. doi: 10.33102/mjsl.vol10no1.345

[ref52] Rahman M. M. Watanobe Y. (2023). ChatGPT for education and research: opportunities, threats, and strategies. Appl. Sci. 13:5783. doi: 10.3390/app13095783

[ref53] Rahmatizadeh S. Kirakowski J. Valizadeh-Haghi S. Taheri M. Tavasoli S. (2024). A combination of three scales for measuring user-perceived usability of a clinical information system: which approach produces the Most informative results? Front. Health Inform. 13:193. doi: 10.30699/fhi.v13i0.569

[ref54] Septiyanti N. D. Luthfi M. I. Darmawansah D. (2024). Effect of Chatbot-Assisted Learning on Students’ Learning Motivation and Its Pedagogical Approaches. Khazanah Informatika: Jurnal Ilmu Komputer dan Informatika, 10, 69–77. doi: 10.23917/khif.v10i1.4246

[ref55] Sharma R. Kumar A. Chuah C. (2021). Turning the blackbox into a glassbox: an explainable machine learning approach for understanding hospitality customer. Int. J. Inform. Manag. Data Insights 1:100050. doi: 10.1016/j.jjimei.2021.100050

[ref58] Syahmani S. Hafizah E. Sauqina S. Adnan M. Ibrahim M. (2021). STEAM Approach to Improve Environmental Education Innovation and Literacy in Waste Management: Bibliometric Research. Indonesian Journal on Learning and Advanced Education (IJOLAE), 3, 130–141. doi: 10.23917/ijolae.v3i2.12782

[ref56] Thamilarasan Y. Raja Ikram R. R. Osman M. Salahuddin L. Bujeri W. Y. W. Kanchymalay K. (2023). Enhanced system usability scale using the software quality standard approach. Eng. Technol. Appl. Sci. Res. 13, 11779–11784. doi: 10.48084/etasr.5971

[ref57] Vlachogianni P. Tselios N. (2022). Perceived usability evaluation of educational technology using the system usability scale (SUS): a systematic review. J. Res. Technol. Educ. 54, 392–409. doi: 10.1080/15391523.2020.1867938

[ref59] Yanfi Y. Udjaja Y. Sari A. C. (2020). User’s demographic characteristic on the evaluation of gamification interactive typing for primary school visually impaired with system usability scale. Adv. Sci. Technol. Eng. Syst. J. 5, 876–881. doi: 10.25046/aj0505107

[ref60] Zara M. Y. (2022). Islamic patriotism in general Sudirman comic strips of Suara Muhammadijah magazine (1966-1967). Stud. Islamika 29, 305–332. doi: 10.36712/sdi.v29i2.19588

[ref61] Zhou L. Gao J. Li D. Shum H.-Y. (2020). The design and implementation of XiaoIce, an empathetic social Chatbot. Comput. Linguist. 46, 53–93. doi: 10.1162/coli_a_00368

